# Kinesiophobia in heart disease: ‘it is part of the process’ or is it? Perspectives from cardiac rehabilitation professionals – a qualitative study in healthcare settings

**DOI:** 10.1136/bmjopen-2025-101393

**Published:** 2025-12-05

**Authors:** Amy Jane Jacob, Abraham Samuel Babu, Sebastian Padickaparambil

**Affiliations:** 1 Department of Clinical Psychology, Manipal College of Health Professions, Manipal Academy of Higher Education - Manipal Campus, Manipal, India; 2 Barnet Crisis Resolution and Home Treatment Team, Barnet Enfield and Haringey Mental Health NHS Trust, London, UK; 3 Department of Physiotherapy, Manipal College Of Health Professions, Manipal Academy of Higher Education - Manipal Campus, Manipal, India

**Keywords:** Cardiovascular Disease, MENTAL HEALTH, QUALITATIVE RESEARCH

## Abstract

**Objectives:**

To explore cardiac rehabilitation (CR) professionals’ perspectives on kinesiophobia in patients with cardiovascular diseases. This study aims to understand the perspectives of healthcare professionals (HCPs) regarding their perceptions, assessments and management of kinesiophobia.

**Design:**

A qualitative descriptive study using in-depth interviews and thematic analysis.

**Setting:**

The study was carried out through online interviews at a university teaching hospital in South India.

**Participants:**

HCPs involved in CR, from around the world, were invited to participate through advertisements on social media and through professional forums. 12 HCPs, including CR nurses (n=1), CR physicians (n=1), cardiac surgeons (n=1), cardiac electrophysiologists (n=1), rehabilitation physicians (n=1), cardiologists (n=2), exercise physiologists (n=2) and physiotherapists (n=3), agreed to participate.

**Interventions:**

Not applicable (qualitative study without interventions).

**Primary and secondary outcome measures:**

Qualitative data collected through in-depth interviews focused on HCP perceptions regarding kinesiophobia and its assessment, management and awareness within CR.

**Results:**

Thematic analysis generated 337 codes, which formed seven subthemes: the perceived burden of kinesiophobia, reasons for kinesiophobia, HCP experiences with kinesiophobia, methods of assessing kinesiophobia, management strategies, reasons why kinesiophobia is overlooked and the importance of promoting awareness of kinesiophobia.

**Conclusions:**

CR professionals recognise kinesiophobia as a significant issue among patients with heart disease but do not recognise the term or perceive it as a separate condition; instead, they view it as part of the overall clinical presentation. There is a strong need to advocate for early recognition and assessment of kinesiophobia and for the development of structured management strategies and its inclusion into CR programmes to improve patient outcomes during recovery.

**Trial registration number:**

The study was prospectively registered in the Clinical Trial Registry of India (CTRI/2022/05/042502). This study received approval from the Kasturba Medical College and Kasturba Hospital Institutional Ethics Committee-2 (Student Research) with reference number IEC2:13/2022.

**STRENGTHS AND LIMITATIONS OF THIS STUDY**

- This study explored perspectives on kinesiophobia from healthcare professionals in cardiac rehabilitation.
- Participants in this study are from a global platform that provides an international perspective on addressing kinesiophobia in cardiac rehabilitation.
- A key limitation was the lack of psychological professional representation within cardiac rehabilitation and hospital settings.

## Introduction

 In addition to experiencing physical and functional difficulties, individuals with heart disease also experience psychological problems such as anxiety, depression, hostility and social isolation.^1^,^3^ Research has shown that individuals with heart disease, particularly those who have experienced a major cardiac event, often engage in fear-driven behaviours, such as hesitating to exercise or participate in physical activities, which are considered psychological reactions to such an event.^1 4 5^ However, it is essential to differentiate expected presentations from irrational and excessive fear of movement following a significant cardiac event, as this distinction can highlight the presence of a phenomenon known as kinesiophobia.

Kinesiophobia is defined as “an excessive, irrational and debilitating fear of movement and activity, resulting from a feeling of vulnerability to painful injury or reinjury”,^3^ which can result in physical dormancy, disability and depression.^1^ Kinesiophobia occurs in 20% of patients with Coronary Artery Disease (CAD) after 6–10 months of an acute episode.^6^ Kinesiophobia is associated with increased self-reported disability and decreased physical performance and may predict future occupational disability.^7^ Kinesiophobia also affects participation in cardiac rehabilitation (CR), as demonstrated by a lack of physical exercise, lower physical activity levels, reduced aerobic exercise capacity and poorer health-related quality of life. Kinesiophobia is associated with conditions such as frozen shoulder, hypervigilance during physical exercise and physiological changes, including increased heart rate.^8 9^ Thus, kinesiophobia should be considered an important aspect when formulating the psychosocial domains of cardiovascular diseases.^10^

Kinesiophobia has been widely explored in patients with chronic pain, fibromyalgia, upper extremity disorders and osteoarthritis, and in the general population.^10^,^14^ Nevertheless, it has rarely been studied in individuals with heart disease. With psychological interventions being a core component of CR, professionals might benefit from incorporating concerns such as kinesiophobia into their formulation of patients’ needs. However, there is limited research on healthcare professionals’ (HCPs) perceptions of kinesiophobia. Therefore, this study aimed to explore the perceptions of kinesiophobia among CR professionals.

## Methods

This qualitative study used in-depth interviews with CR professionals between February 2022 and September 2022. The study is reported as per the Consolidated Criteria for Reporting Qualitative Research (COREQ) guidelines.^15^ This study received approval from the Kasturba Medical College and Kasturba Hospital Institutional Ethics Committee-2 (Student Research) with reference number IEC2:13/2022 and the Institutional Research Committee. Additionally, this study was registered prospectively in the Clinical Trial Registry India (Reference number: CTRI/2022/05/042502). Since the study did not purposively recruit participants from specific centres around the world, and a snowball sampling method was used, no site-specific ethics approvals were required for this study. Furthermore, the aim of the study was not to compare geographic differences in views on this topic.

Through advertisements and the sharing of information on professional forums and social media platforms (eg, LinkedIn, Facebook and Instagram), the investigators (AJJ, ASB, SP) reached out to experts in the field of CR to invite their participation. They were eligible to participate if they had a minimum of 5 years of clinical experience and were currently involved in caring for patients recovering from heart disease and major cardiac events. Potential participants were also identified from published literature search. Once the participants were identified for the current study, they were contacted via email by the principal investigator (AJJ). All participants meeting the inclusion criteria provided written informed consent before the interviews. The principal investigator (AJJ) recorded the data, removed identifiers and assigned a unique code to each participant to ensure their anonymity.

### Data collection

Once the participants were identified by the investigators (AJJ, ASB, SP), they were invited by the principal investigator (AJJ) via email to complete the consent form and provide a list of potential dates and times, along with their time zones, to schedule the interviews on online platforms (Zoom and Microsoft Teams). The interviews were conducted from June to October 2022 by the principal investigator (AJJ), who has previous training and experience in qualitative research. Each interview in the current study lasted approximately 45–60 min and was conducted online in a room free from noise and distractions, where only the interviewer was present. At the start of each interview, the principal investigator (AJJ) explained the purpose of the study, assured participants of confidentiality and collected demographic details. All interviews were audio-recorded and video-recorded, transcribed and transliterated offline to ensure the accuracy of the information. Data collection was stopped when the investigators recognised similar codes and subthemes and concluded that no new themes were being generated from additional interviews. No repeat interviews were conducted nor were there any participant dropouts.

### Interview guide

A semistructured proforma was used to collect the sociodemographic details of the participants. The investigators (ASB, SP, AJJ) constructed a qualitative interview guide (see online supplemental file 1 – Interview guide) based on existing research on professionals’ awareness of kinesiophobia, which contained six primary questions (open-ended). The questioning strategy was responsive to each participant, and the interviewer used probes to clarify comments and elicit in-depth explanations.

### Data analysis

**Figure 1:**
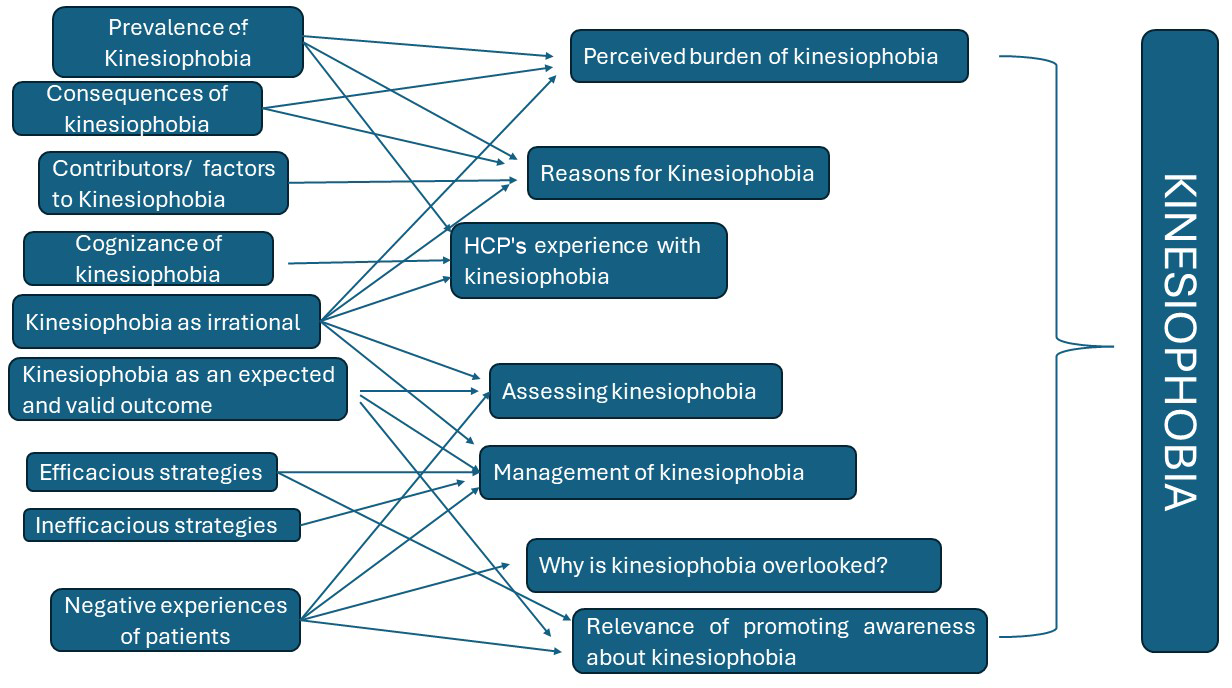
Perspectives of healthcare professionals on kinesiophobia.

A thematic analysis was used in the study. The interviews were audio-recorded and video-recorded, played back and transcribed offline to ensure the accuracy of the information. The verified transcripts were read systematically and analysed using a six-phase thematic analysis.^16^ These steps involved familiarising with the data (transcripts) and generating initial codes with NVivo software (trial version) to organise the data. Statements that illustrated the essence of each code were highlighted, and numbers were assigned to locate the codes within the transcripts. The initial codes were subsequently reviewed by another investigator (SP). Finally, all the authors (AJJ, ASB, SP) checked and discussed the codes. After the entire dataset was coded, a code list was generated in NVivo (trial version) and verified with the transcripts. The codes were grouped into potential subthemes for analysis and presentation and later revised to form themes via an inductive process. After 10 transcripts were coded, the investigators had nearly reached data saturation, as they began to observe repetitive codes and subthemes. The investigators also coded two more transcripts to confirm data saturation and noted that no new themes emerged. The analysis of the data revealed a total of 337 codes. These codes were then combined into seven subthemes (figure 1).

The principal investigator (AJJ) acknowledges that their psychological background and personal beliefs may have influenced their interactions with, and interpretations of, the interviews. To mitigate bias, the principal investigator (AJJ) engaged in self-reflection, used supervision from the senior investigators (SP and ASB) and prioritised participants’ views in the research. Additionally, the principal investigator (AJJ) maintained a detailed audit trail to ensure dependability and employed triangulation to enhance confirmability. The transcripts were not returned to the participants for comments or feedback.

## Results

12 CR professionals, with a mean experience of 15.5±10.2 years worldwide, participated in the study (table 1). Figure 1 illustrates HCPs’ perspectives on kinesiophobia. Quotes supporting each theme are reported in online supplemental table 1.

**Table 1:** Sociodemographic details of the interviewed practitioners

Participant ID	Occupation	Country
ID01	Exercise physiologist	USA
ID02	Doctor	India
ID03	Chest physiotherapist	Bangladesh
ID04	Cardiac rehabilitation nurse	Australia
ID05	Assistant professor – Department of Cardiology	India
ID06	Physical medicine and rehabilitation specialist (physiatrist)	Spain
ID07	Physical therapist	The Netherlands
ID08	Chief medical officer/cardiologist	India
ID09	Cardiovascular specialist (surgeon)	India
ID10	Clinical professor – exercise physiologist	USA
ID11	Cardiac electrophysiologist	India
ID12	Cardiac physiotherapist	Spain

### Subtheme 1: Perceived burden of kinesiophobia

Most professionals agreed that a fear of movement was expected and not considered unusual or burdensome. However, interestingly, kinesiophobia was not addressed by most of the participants.

It is, oh, it is very common, and it is not something that surprises me in any individual who comes to a cardiac rehab program*. –* ID004

I have seen most of them; they usually don’t have a first or second follow-up. Maximum, they will have some problem, but after that their pain scores reduce, their activity where they get back to their routine activities. – ID009

to be honest, even though it is a common occurrence. This is just a natural part of recovery. – ID010

Yeah, yes, it is unrealistic, but the experience is so overwhelming that they know they somehow subconsciously feel that I’m safest when I’m absolutely still. Therefore, it is justified, but that is where counsellors will reassure them, that you know. Therefore, you need to know the science of rehabilitation. To help them with early movements. – ID08

…yes, it would be helpful to know if someone truly had unusual fears and things, but I think we assume that most people have some fear, right? That’s natural. – ID01

### Subtheme 2: Reasons for Kinesiophobia

Various factors and patient beliefs drive kinesiophobia, which encompasses fears of re-injury, subsequent heart attacks and mortality, all of which are heightened by physical sensations. Concerns about displacing implanted devices further contribute to this fear, with family members significantly influencing patients’ activities and prognosis. The intensity of kinesiophobia correlates with medical conditions, the acuteness of the procedure and specific cardiac interventions. Among behavioural and psychological factors, awareness of the benefits of physical activity and exercise motivation are prominent, whereas aspects such as self-efficacy and social constraints are less emphasised in the data. Practitioners’ insights reinforce the impact of these variables on kinesiophobia.

I feel that they are fearful of pain, and they are fearful of dying. – ID004.

Some patients really are afraid because they have certain sensations that make them afraid, while other patients do not have those sensations, but they’re still afraid. In addition, you have people who have a lot of distressing body signals. – ID007

### Subtheme 3: HCPs’ experiences with kinesiophobia

HCPs recognise the fear of movement but may not identify kinesiophobia as a separate phenomenon. Awareness varies, with insights gained through clinical experience or formal training. HCPs acknowledge a high prevalence of fear of movement, especially during the acute phase. HCPs perceive kinesiophobia to be influenced by factors such as the type of medical procedure and the acuteness of cardiac issues. The observed psychosocial impacts include effects on early mobilisation, daily functioning, occupation and marital dynamics. Negative experiences contributing to kinesiophobia include postoperative distress, physical complications and psychological factors such as anxiety and fear of trauma. Perspectives on kinesiophobia differ among HCPs, with some placing emphasis on education and behavioural techniques. This study highlights the importance of targeted screening and interventions.

I think it’s interesting that you brought up this terminology for this fear of exercise. It is not something I have thought much about other than when I think about it. I have worked in this area, you know, for 12 years. I didn’t know what that word was. I had googled it. – ID04.

Yes, they do have a lot of psychosocial, psychosomatic and behavioural problems that come from their…either their knowledge of heart disease or lack of knowledge of heart disease. – ID08

I used a different way to frame kinesiophobia, so you have subclinical levels. You have mild symptoms, you have moderate symptoms, and you have severe symptoms. mild and moderate levels are truly quite common. – ID07

It was taught to us…but with different populations. Therefore, with patients with low back pain, for instance, was there not much attention given to cardiac rehabilitation at that time, but it is definitely an important part of education for physical therapists. – ID07

### Subtheme 4: Assessing kinesiophobia

Assessments and screening are vital for establishing baseline functioning, identifying risk factors and evaluating positive rehabilitation outcomes. Physiological assessments include the walk test, Borg scale administration, heart rate measurement during exercise, and exercise capacity tests. Psychological screening covers depression, stress, anxiety, kinesiophobia and quality of life, which are crucial for addressing mental health issues and optimising rehabilitation benefits. The participants emphasised the importance of physiological assessments, using objective measures to reassure hesitant patients. Suggestions for a holistic evaluation included disease status, medication compliance, diet and mental health screening at regular intervals. The study highlights the importance of psychological assessments and screening, identifying commonly used assessments that evaluate both physiological and psychological components. Notably, the infrequent mention of the Tampa Scale in one interview highlights the limited measurement and incorporation of kinesiophobia into treatment.

We have recently validated the Tampa Scale for Kinesiophobia. Therefore, we are using that now in our digital program. Therefore, patients can fill it in after hospital discharge… and then fill it again when they start physical rehabilitation and at the end. However, it is its part of, multiple assessment tools we use. Therefore, we also use the hospital anxiety and depression scale; we also monitor quality of life, etc. Therefore, it is part of a battery of multiple questions. – ID07

Ask direct questions to the patient at discharge, at first follow-up. We have a checklist of different aspects, at least the major headings, as to one’s medication compliance, dietary compliance, and, of course, disease status, which we are assessing. Therefore, we can have a checklist for diet, we can have a checklist for mental health, you know which can be filled out, you know, even a counsellor or nursing professional when the patient enters or if the patient is educated, he himself can fill out that checklist and then we immediately get to know what other aspects he needs and then we can redirect treatment accordingly. – ID05

### Subtheme 5: Management of kinesiophobia

This study examined strategies used by HCPs to manage cardiovascular patients, with a focus on kinesiophobia. Standardised approaches included educating patients, validating concerns and using empathetic listening. Behavioural change models such as motivational interviewing and graded exposure were recommended. Motivating patients through education, assessing motivation and using objective measures such as walk tests were emphasised. Group settings were suggested to promote observational learning and enhance patient empowerment. Gentle initiation of physical activities was preferred over coercion. The involvement of mental health professionals in employing psychological strategies was suggested (ie, graded exposure, relaxation techniques, mindfulness, modelling, observational learning, family intervention, psychoeducation and motivational interviewing). The study highlighted the role of a robust patient-practitioner relationship. Additional strategies included yoga, spiritual support and collaboration with multidisciplinary teams.

We also try to use the graded exposure technique. Therefore, during exposure to activity, you always reflect with patients on how they feel and if they have any distressing body signals. – ID07.

…the fear that they have is not rational. It’s an irrational fear, so teaching them not to be afraid is truly important, and we deal with almost every patient. – ID10.

Come and see that, that’s OK, ‘hey, look at that guy, he had the same bypass surgery you did a week ago or a month ago. Look what he’s doing… let’s walk at your pace. Let us start this at your pace and realise that you will be monitored. We will have EKG electrodes or check your blood pressure. – ID01.

But then what we’ve also seen is if you start giving them confidence, talking to them, making them take baby steps, baby steps, become bigger steps, and over time their confidence grows. It usually does not end up being a long-term problem, but yes, you need to address it. You can't ignore it. – ID02

You need to speak with their spouse…to get them to understand because the spouse often has a major influence on the patient and whether they comply – ID10.

### Subtheme 6: Why is kinesiophobia overlooked?

Most HCPs recognise a fear of movement in their patients but do not attribute it specifically to kinesiophobia. They consider fear of movement after a significant cardiac event to be normal, viewing it occasionally unusual or irrational. However, they acknowledge that its intensity may warrant intervention in the acute stage. HCPs in the study describe kinesiophobia on a range rather than an all-or-none phenomenon, with the intensity of fear guiding its management. Although addressing kinesiophobia is questionable, the study suggests a favourable inclination toward managing it effectively. Some HCPs argue against universal screening for kinesiophobia and instead focus on high-severity cases. While some HCPs rejected the idea of further professional training on kinesiophobia, the majority acknowledged the need to explore it among patients in the future.

…let’s say you had a heart attack, or you have gotten a stent … you worry when this is going to happen again to me, right? So gonna linger, I think, for some… for most people, for some time. I do not think it should linger for months. However, it should, you know, it is supposed to be normal to worry about when it’s going to happen again. – ID01

See, you cannot put common sense into people. It is not a skill that you will learn. See, you see a patient; you know how to treat him. Therefore, there are two things; including it in the curriculum will not make any difference. – ID09.

Umm, well, it isn’t in our curriculum, and it isn’t definitely in the curriculum of the physicians either. The positions that, as it is, they receive little enough training in prevention and rehabilitation. The focus is always on treatment. – ID10.

I do not think I have come across anybody I would label this person, owing to a fear of movement. Maybe some people might…we go by the fact that some people are slightly more enthusiastic to exercise. Some people are a little bit less. The people may be a little less; they fear movement, but we never considered that a term, right? Or do you think of that as a label? So OK, it’s possible that it is there, but I don’t know of it. – ID02

### Subtheme 7: Relevance of promoting awareness of kinesiophobia

Debates surround the approach to addressing kinesiophobia in the CR. Nevertheless, experts recommend increasing awareness of kinesiophobia among professionals. Some strategies suggested by HCPs are workshops, training in health behaviour and psychosocial issues, and assigning a dedicated team member for screening and management. Educational strategies such as incorporating kinesiophobia into curricula and providing early exposure to rehabilitative care are highlighted. The findings underscore the importance of research to encourage professionals to screen, assess and manage kinesiophobia in CR, suggesting a biopsychosocial approach to enhance treatment outcomes and improve patient quality of life.

Yes. I think having this topic in our curriculum would be fine. This information is based on scientific evidence. Therefore, it could be better for us. – ID12

Well, we should share knowledge about this. With respect to kinesiophobia and its impact on patients’ lives, professionals should provide clear guidelines on how to work with it. I think that’s the most important. Therefore, not only measuring it, but also screening and observing it…I think that is the main thing, and being sensitive about the phase that the patient is in. Especially in the first phase of cardiac rehabilitation, I think it is truly important that there is more attention moving forward during hospital discharge or weeks after. That is also when patients realise what they have gone through, and they often have this, you know, feeling of worry and anxiety. That is also when they should be given more attention. Therefore, it also has to do with the timing, I think. – ID07

So just bringing it out in the open by sensitising all the stakeholders would make the biggest impact in the initial phases. – ID05

### Patient and public involvement

Patients and/or public were not involved in the design, conduct, reporting or dissemination plans of this research. The study participants were HCPs who provided informed consent.

## Discussion

The current study explored HCPs’ perspectives on kinesiophobia in the context of CR. Previous research on kinesiophobia has examined it in other health conditions and has focused on the patient perspective. In contrast, the current study focuses on how HCPs conceptualise and respond to this phenomenon. Participants recognised kinesiophobia as a fear of movement or exercise attributed to fear of reinjury, pain, recurring cardiac events, displacement of implants and even death, which is consistent with earlier studies.^17^ However, awareness of kinesiophobia as an independent construct was variable; some HCPs perceived it as an expected and valid reaction after a major cardiac event rather than an unusual problem, while others viewed it as a separate construct on the basis of severity. Some HCPs questioned the need for universal screening for kinesiophobia and preferred to focus on severe presentations, whereas others supported a routine assessment and management. The participants in the study suggested various physiological and psychological measures used during CR (indicating a biopsychosocial model). Some of the measures mentioned were the walk test, administration of the Borg scale, measurement of heart rate during exercise and exercise capacity, respiratory test, hand dynamometer, Beck’s Depression Inventory (BDI), Hospital Anxiety and Depression Scale (HADS), Patient Health Questionnaire-9 (PHQ-9) and Tampa Scale of Kinesiophobia.

The HCPs in the present study reported that kinesiophobia is more prevalent during the acute phase of treatment (Intensive Care Unit [ICU], inpatient care) and less common in subsequent phases of CR. Nevertheless, others reported a high level of kinesiophobia over time.^18^ The discrepancy noted in the study may be influenced by programme structure, early-phase rehabilitation and the availability of psychological support. The participants acknowledged that kinesiophobia leads to reduced CR participation and poor prognosis, which aligns with previous evidence.^17^ This is highlighted in a study where kinesiophobia was found to impede the initiation of cardiac rehabilitation, where even a one-point increase on the Tampa Scale for Kinesiophobia reduced odds of CR initiation by approximately 8%. This reduction was attributed to cardiac anxiety, social complexities, education level and self-efficacy.^19^ Furthermore, in patients who have experienced myocardial infarction, high kinesiophobia was associated with poor physical activity levels, lower quality of life, and higher anxiety and depression.^20 21^

The current study identified specific educational and clinical strategies that participants recommended. The participants identified the need to increase awareness of kinesiophobia in professional training, incorporate kinesiophobia in CR curricula for relevant professionals, and increase the representation of psychologists in multidisciplinary teams (MDTs) within CR settings. However, task sharing among professionals does occur.^22^,^26^ The practical implications of this study include increasing awareness of kinesiophobia in MDT settings and developing treatment guidelines for non-psychology professionals to empower them to deliver psychologically informed care. CR experts, including nurses, physiotherapists, cardiac electrophysiologists, rehabilitation physicians, exercise physiologists, cardiologists, CR physicians and cardiac surgeons, are involved in formulating and supervising safe and graded levels of physical activity. The CR experts will be well-positioned to apply behavioural strategies to manage kinesiophobia, help patients build confidence to gradually engage in physical activities and exercise, and provide a validating and empathetic environment.^27 28^

The clinical management strategies include standardised approaches such as a gentle approach to movement, psychoeducation, family involvement and the use of objective measures (heart rate monitors, oxygen levels) to build confidence while exercising. The participants also highlighted behavioural strategies such as motivational interviewing, graded exposure, relaxation techniques and observational learning in group settings. Recent Behavioural Exposure for Interoceptive Tolerance (BE-FIT), an Acceptance and Commitment Therapy (ACT)-informed exposure-based intervention that focuses on accepting bodily sensations rather than eliminating fear, appears to hold promise as a potential strategy.^29 30^ HCPs recommended a strong patient-practitioner relationship and collaboration with MDTs. The findings of the present study align with behavioural and psychological strategies supported in other clinical contexts, such as knee arthroplasty and lumbar dysfunction.^27 28^ The psychological strategies noted in the current study reflect the biopsychosocial model, which emphasises the connection between physical recovery, emotional responses and social support.

The current study noted that variation in HCP perspectives may reflect constraints in health systems across different cultures and geographical locations, HCP attitudes toward movement after a significant illness, differences in training, and systemic prioritisation of physical rehabilitation over psychological rehabilitation. These factors may hinder the recognition and management of kinesiophobia. The study also noted the limited presence of psychologists in CR settings.^31^

### Limitations

The current study’s professional representativeness is limited by the difficulty in recruiting psychologists with CR experience and only a few specific professional groups (nurses, physiatrists). Furthermore, participation bias is possible, as those with an interest in psychosocial aspects of CR may have been more likely to respond. Variability in how the participants understood and defined ‘kinesiophobia’ could also have influenced the data. Information on tools for assessment of kinesiophobia and specific interventions was not explored in detail.

Future studies could assess the prevalence and impact of kinesiophobia across CR phases and focus on quantitative assessment, as the current study was qualitative. Studies could explore the cultural and systemic influences on the recognition and management of kinesiophobia and test the effectiveness of incorporating kinesiophobia into CR curricula for relevant professional stakeholders.

### Conclusion

The study emphasises the importance of recognising kinesiophobia as a distinct phenomenon within CR settings. Time, the CR professionals in the current study demonstrated awareness of fear of movement, but very few formally recognised kinesiophobia and its systemic management. Therefore, it is essential to recognise kinesiophobia through future research, implement it in CR training and curricula, strengthen the biopsychosocial approach in CR, encourage multidisciplinary collaboration and address workforce gaps.

## Supplementary material

10.1136/bmjopen-2025-101393 online supplemental file 1

10.1136/bmjopen-2025-101393 online supplemental file 2

## Data Availability

Data are available upon reasonable request.
