# Supplementary material for: Kinesiophobia in heart disease: ‘it is part of the process’ or is it? Perspectives from cardiac rehabilitation professionals – a qualitative study in healthcare settings
Source: BMJ Open. 2025 Dec 5;15(12):e101393. doi: 10.1136/bmjopen-2025-101393 (PMC12684105; doi:10.1136/bmjopen-2025-101393)
Supplement: online supplemental file 1 [file bmjopen-15-12-s001.docx]

**INTERVIEW GUIDE**

Good morning/afternoon/evening. I am XXXX from the Dept. of Clinical Psychology. Thank you for agreeing to be a part of this study. Before we begin, may I confirm that you agree to be a part of this study and consent to being recorded?

Thank you.

**1. To begin with, could you please tell me about your work in cardiac rehab?**

**PROBES**

a. How long have you been working?

b. How do you find your work?

c. Would you say your work in CR is always exciting and there is something new to be learnt from each patient?

i. Could you elaborate?

d. What has been the biggest challenge you have faced?

i. Could you provide more details?

ii. Why did you feel this was a challenge?

iii. Was this something unexpected?

iv. How did you deal with it?

v. How has that changed you in your practice?

e. What would you say is the biggest lesson you have learnt from your patient?

i. How has this has had an impact on you?

**2. Have the problems of your patients prompted you to try something new w.r.t your interventions?**

**PROBES**

a. Could you give me an example or list a few that come to mind?

(If someone mentions fear of movement – go forward to ask more about it)

Cardiac Rehab Professionals’ Perspectives on Kinesiophobia

b. How did these problems make you modify your treatments?

**3. How important do you think it is to explore problems that patients have which impact cardiac rehab?**

**PROBES**

a. Why do you say so?

b. Are there any problems you feel that have not been explored?

i. What are they?

ii. Why do you feel they have not been explored?

iii. Is it really warranted to understand more about it or can we just be content saying “it is logical” or “it is expected”?

c. Do you feel CR professionals fail to explore certain problems, which they think are “expected”?

i. Could you give me an example?

d. Have you come across a problem that you felt could have been better handled by another CR member?

i. What was it?

ii. If the other CR member was not available, how did you manage it?

e. In your experience, have you found the absence of a particular team member to be affecting the way you treat a patient or the outcomes for the patient?

i. How has this situation made you feel professionally?

ii. Did you take any steps to remedy this situation?

iii. In the era of ‘task sharing’ do you think you could take on the role of another team member to deliver CR?

1. Why/why not?

2. How would it make you feel if you felt someone else was

learning your profession?

**4. I gather, many of your patients are afraid to move initially after an acute cardiac event, how often do you see this?**

**PROBES**

a. Do you see this as a major factor affecting your CR that needs to be addressed?

b. How do you deal with this?

c. What are your thoughts on a forceful versus a gentle approach to getting them to move?

d. How would you balance the need to move versus patient’s fear of moving?

Cardiac Rehab Professionals’ Perspectives on Kinesiophobia

e. Have you felt the patient’s fear of moving to be an unrealistic fear?

i. Why/why not?

f. Have you gone about trying to remove their fear of moving?

i. Why/ why not?

ii. What made you want to do this?

iii. How did you do this?

g. Is it important to address this fear?

i. Why?

ii. Do you think this requires support from any other CR team

members?

1. If so, who?

h. Do you think other members in your CR team notice this?

i. Is there any reason for this?

i. Have you noticed this as a barrier to treating your patient?

i. Why/why not?

**5. Do you think in your professional training, this fear of movement was ever addressed?**

**PROBES**

a. How was it covered?

b. Do you think if your training included this, you would be better

equipped at handling patients with a fear of movement?

i. If so, how would you like this to be incorporated into the curriculum?

**6. What would make you more sensitive to the assessment and management of fear of movement?**

**PROBES**

a. Why do you think these would?

b. What would you suggest as ways to make CR professionals more sensitive to this?

i. How would you go about this?

Is there any thing else you would like to share that you think we have missed out on this topic

Thank you .
