# Supplementary material for: Kinesiophobia in heart disease: ‘it is part of the process’ or is it? Perspectives from cardiac rehabilitation professionals – a qualitative study in healthcare settings
Source: BMJ Open. 2025 Dec 5;15(12):e101393. doi: 10.1136/bmjopen-2025-101393 (PMC12684105; doi:10.1136/bmjopen-2025-101393)
Supplement: Supplementary file 3 [file bmjopen-2025-101393.reviewer_comments.pdf]

PEER REVIEW HISTORY

BMJ Open publishes all reviews undertaken for accepted manuscripts. Reviewers are asked to complete a checklist review form and are provided with free text boxes to elaborate on their assessment. These free text comments are reproduced below.

ARTICLE DETAILS

Title (Provisional)

‘Kinesiophobia in Heart Disease: 'It Is Part of the Process' or Is It? Perspectives from Cardiac Rehabilitation Professionals—A Qualitative Study in Healthcare Settings

Authors

Jacob, Amy Jane; Padickaparambil, Sebastian; Babu, Abraham Samuel

---

VERSION 1 - REVIEW

**Reviewer**	**1**
**Name**	**White, David A.**
**Affiliation**	**Children's Mercy Kansas City Adele Hall Campus, Ward Family Heart Center**
**Date**	**19-Jun-2025**
**COI**	**None**

Thank you for the opportunity to review the manuscript titled “Kinesiophobia in heart disease: “It is part of the process”, or is it? Perspectives from Cardiac Rehabilitation professionals”. The qualitative study aims to explore the knowledge of kinesiophobia (KP) and perspectives of KP in CR professionals. My expertise is not qualitative research and unfortunately, I will not be able to provide much feedback on the methodology used in this study. However, I have particular interest and expertise in KP and its application in patients with cardiac disease.

Overall, I found the manuscript to be well written and mostly clear. However, I found several instances where the writing became unclear, and I found myself re-reading sentences to understand what the author(s) were trying to convey. In a scientific paper, someone should be able to pluck any individual sentence from anywhere in the paper and place it on a blank piece of paper and it would be its own complete thought. The sentences that I found confusing and should be re-written are listed below:

Introduction:

Sentence: “This could lead to fear and avoidance behaviours, which are normal psychological reactions to this event (1,5).” It is unclear what ‘this event’ is referring to. Also, the authors should consider replacing ‘and’ with ‘driven’.

Sentence: “These negative experiences affect cardiac rehabilitation (CR) participation, which is essential to improving psychological factors(8,9).” What negative experiences is being referred to? And in what way (direction) do the negative experiences affect CR?

Methods:

Sentence: “They were requested to participate in the research study by e-mail and an attached flyer containing details of the study, and they were also informed regarding the researcher’s interest in the topic.” Who is ‘they’ in this sentence? Please clarify.

Sentence: “To mitigate bias, they engaged in self-reflection and supervision and prioritised participants' views in the research. Additionally, they maintained a detailed audit trail for dependability and used triangulation to enhance confirmability.” In these sentences, it is unclear who ‘they’ is referring to.

Discussion:

Sentence: “The participants strongly advised additional training and inclusion of kinesiophobia in the curriculum.” What curriculum is this sentence referring to?

Sentence(s): “Fundamental awareness of kinesiophobia as fear of movement gained through clinical experience is present among professionals, and the study additionally identified the requirement to include it as part of the educational curriculum of health professionals involved in cardiac rehabilitation. The professionals were not aware of it as an independent phenomenon.” What is ‘it’?

Sentence: “Yet, physical and psychological strategies were commonly incorporated to facilitate participation in CR.” I do not think this is a clear or complete sentence.

Other considerations for the authors:

Abstract:

Sentence: “The lack of trained psychologists within CR teams to tackle kinesiophobia is a significant concern, not to mention the gross underutilization of CR.” The authors should consider re-writing this sentence, using more sophisticated language such as ‘address’ rather than ‘tackle’. Also, the incorporation of “…not to mention the gross underutilization of CR” seems like conjecture from the author which does not align with the purpose of the paper or the data collected.

Introduction:

Minor comment: In the sentence “With psychological interventions being a core component of CR, CR professionals need to understand other psychological concerns like kinesiophobia that can be seen in those with CAD” try to avoid using two abbreviations back-to-back.

Methods:

“Eligible participants were contacted via email. All participants meeting the inclusion criteria provided written informed consent before the interviews.” What was the inclusion criteria for this study? Were there more factors than requiring 5 years of experience? Please clarify.

“Participants were identified and communicated through the investigators (AJJ, ASB, SP).” This is a confusing sentence. Please consider re-writing.

Would it be possible to provide a copy of the interview guide as an appendix?

“The initial codes were aimed at addressing the respective research questions of the principal investigator (AJJ).” This is confusing. This is a prospective study, thus, it is unclear what retrospective research question the authors are referring to. Was this study part of another investigation that had different aims?

Results:

Were all the HCPs from adult CR facilities? Were there any pediatric CR experts included? If this was only in adult CR, the authors should clarify that in the methodology.

“The theme of kinesiophobia will be elaborated on in detail in this paper.” This is confusing. I am not sure how this sentence contributes to the paper. I was under the impression that this study focused on KP, but after reading this sentence, I went back as I thought I missed something.

Under “3.6 Subtheme 6: Why is kinesiophobia overlooked?” the second sentence uses ‘FOM’ as an abbreviation for fear of movement, but that abbreviation is not used anywhere else in the paper. In the same respect, there are places throughout the manuscript where cardiac rehabilitation is abbreviated as ‘CR’ and other places where it is written out. Please make consistent.

Discussion:

Sentence: “The current study attempted to understand the HCP's perspective on kinesiophobia under the theme of kinesiophobia.” It may be that I am not familiar enough with qualitative research, but this sentence seems confusing. Is it necessary to note that this study on KP uses KP as its theme? It seems like a redundant sentence.

Sentence: “There seems to be a certain amount of discrepancy regarding the presentation of kinesiophobia in the preliminary stages of CR in the current data and existing literature.” Please consider revising this sentence to use more sophisticated language. The authors could revise this sentence to say ‘We observed some discrepancy regarding the presentation of….’

“Findings that are original to the study are educational and clinical strategies that can be employed to boost the evaluation of kinesiophobia.” I believe this study presented HCPs perspectives on KP in CR settings. This sentence is an overreach as I did not find any educational or clinical strategies or recommendations presented by the data collected on how KP can be evaluated.

Lastly, it is clear from the interviews that the HCP were not familiar with KP as an established construct. This should not be surprising as KP originated from the chronic pain research and has not been thoroughly applied in other clinical populations. It would be unreasonable to expect CR HCP to be familiar with psychological constructs from fields outside of their own.

**Reviewer**	**2**
**Name**	**Keessen, Paul**
**Affiliation**	**Amsterdam University of Applied Sciences, Faculty of Health**
**Date**	**15-Jul-2025**
**COI**	**I have published several articles about this topic.**

Congratulation with this study. There are several concerns that need to be addressed- before publication.

Overall concern: The authors have some interesting results. These results ask for a more thorough reflection in the discussion section. At the moment the discussion section is too superficial.

Other points:

Abstract:

Purpose: `Furthermore, the views of health care professionals (HCPs) in cardiac rehabilitation (CR) on kinesiophobia and the importance of addressing it were lacking.'

Please use the present tense here.

Methods

The authors state: `Data collection was halted after data saturation, as discussed by the research team. No repeat interviews were conducted..'

How did the authors decide when data saturation was achieved?

The interview guide was not included in the review. Please add the interview guide for full transparency

Data analysis: please provide a reference of the used methodology.

Results

Subthemes: please provide more quotes. The authors describe many factors results which are not supported by quotes. It would help of more quotes were provided. The authors refer to the supplementary table which is very helpful but if might help to add some more in the article itself.

Discussion

The authors state:

`HCPs also claimed that kinesiophobia was more prevalent during the acute phase of treatment (ICU, in-patient) and lesser in subsequent phases of CR (18). Data suggests that kinesiophobia among patients led to reduced participation in the rehabilitation process and affected prognosis(17).'

There is research indicating that kinesiophobia remains stable over time. The authors do not reflect on all the published literature. It might be good to elaborate more on this topic.

The authors state: `Although standardised guidelines require a psychologist to be part of the multidisciplinary team, it has been found that a psychologist may not always be present, and this potentially impacts how specialists may offer psychological care (22). In identifying practitioners for the study, it was observed that the number of psychologists practicing as part of cardiac rehabilitation was slim.'

The authors stress the need for a psychologist. This is indeed important. However, the authors might also want to discuss the role of the physiotherapist in the treatment of kineisophobia.

VERSION 1 - AUTHOR RESPONSE

Section	Modification/Clarification requested	Author's comments	Reference
Abstract	Reviewer 1: Sentence: "The lack of trained psychologists within CR teams to tackle kinesiophobia is a significant concern, not to mention the gross underutilization of CR."<br>The authors should consider re-writing this sentence, using more sophisticated language such as 'address' rather than 'tackle'. Also, the incorporation of "…not to mention the gross underutilization of CR" seems like conjecture from the author which does not align with the purpose of the paper or the data collected.	We appreciate this suggestion. We have removed the sentence and have incorporated this point in the discussion section instead of the abstract.	

Introduction	Reviewer 1: This could lead to fear and avoidance behaviours, which are normal psychological reactions to this event (1,5).” It is unclear what ‘this event’ is referring to. Also, the authors should consider replacing ‘and’ with ‘driven’.<br><br>However, I found several instances where the writing became unclear, and I found myself re-reading sentences to understand what the author(s) were trying to convey. In a scientific paper, someone should be able to pluck any individual sentence from anywhere in the paper and place it on a blank piece of paper and it would be its own complete thought. The sentences that I found confusing and should be re-written are listed below:	We have edited the sentence and incorporated the suggestions made by the reviewer. We have also clarified the sentence as per the reviewer’s advice for the manuscript in general.	Page number: 4<br>Line(s): 4-9<br>Research has shown that individuals with heart disease, particularly those who have experienced a major cardiac event, often engage in fear-driven behaviours, such as hesitating to exercise or participate in physical activities, which are expected to be psychological reactions to such an event. (1,4,5). However, it is essential to differentiate expected presentations from irrational and excessive fear of movement following a significant cardiac event, as this distinction can highlight the presence of a phenomenon known as kinesiophobia.
Introduction	Reviewer 1: However, I found several instances where the writing became unclear, and I found myself re-reading sentences to understand what the author(s) were trying to convey. In a scientific paper, someone should be able to pluck any individual sentence from anywhere in the paper and place it on a blank piece of paper and it would be its own complete thought. The sentences that I found confusing and should be re-written are listed below:	We have clarified the statement: A study has shown that kinesiophobia occurred in 20% of patients with CAD after 6-10 months of an acute episode(6).	Page number: 4<br>Line: 11-12<br>Kinesiophobia occurs in 20% of patients with CAD after 6–10 months of an acute episode(6)
	Reviewer 1: These negative experiences affect cardiac rehabilitation (CR) participation, which is essential to improving psychological factors(8,9).” What negative experiences is being referred to? And in what way (direction) do the negative experiences affect CR?	We have revised the statement to clarify the negative experiences and how CR is affected.	Page number: 4<br>Line(s): 14-18<br>Kinesiophobia also affects participation in CR, which is demonstrated by a lack of physical exercise, lower physical activity levels, reduced aerobic exercise capacity, and poorer health-related quality of life.

			Kinesiophobia is associated with conditions such as frozen shoulder, hypervigilance during physical exercise and physiological changes, including increased heart rate. (8,9).
	Minor comment: In the sentence “With psychological interventions being a core component of CR, CR professionals need to understand other psychological concerns like kinesiophobia that can be seen in those with CAD” try to avoid using two abbreviations back-to-back.	We have avoided using two abbreviations back-to-back and edited the sentence.	Page number: 4<br>Line(s): 22-23<br>With psychological interventions being a core component of CR, professionals might benefit from incorporating concerns such as kinesiophobia in their formulation of patients’ needs.
Methods	Reviewer 1: They were requested to participate in the research study by e-mail and an attached flyer containing details of the study, and they were also informed regarding the researcher’s interest in the topic.” Who is ‘they’ in this sentence? Please clarify.	We have clarified who ‘they’ are in the sentence.	Page: 5<br>Line(s): 19-22<br>Once the participants were identified by the investigators (AJJ, ASB, SP), they were invited by the principal investigator (AJJ) via email to complete the consent form and provide a list of potential dates and times along with their time zones to schedule the interviews on online platforms (Zoom and Microsoft Teams).
	Reviewer 1: To mitigate bias, they engaged in self-reflection and supervision and prioritised participants' views in the research. Additionally, they maintained a detailed audit trail for dependability and used triangulation to enhance confirmability.” In these sentences, it is unclear who ‘they’ is referring to		Page: 7<br>Line(s): 1-5<br>The principal investigator (AJJ) acknowledges that their psychological background and personal beliefs may have influenced their interactions with, and interpretations of, the interviews. To mitigate bias, the principal investigator (AJJ) engaged in self-reflection, utilised supervision from the senior investigators (SP and ASB), and prioritised participants' views in the research. Additionally, the

			principal investigator (AJJ) maintained a detailed audit trail for dependability and used triangulation to enhance confirmability. The transcripts were not returned to the participants for comments or feedback.
	Reviewer 1: Eligible participants were contacted via email. All participants meeting the inclusion criteria provided written informed consent before the interviews."<br>What was the inclusion criteria for this study? Were there more factors than requiring 5 years of experience? Please clarify.	The inclusion criteria has been added and the statement has been edited.	Page: 5<br>Line: 9-15<br>Through purposive sampling, the investigators (AJJ, ASB, SP) identified experts in the field of CR with a minimum of five years of clinical experience with adults who are currently involved in caring for patients recovering from heart disease and major cardiac events. The participants for the current study were identified and invited to participate through a literature search, online advertisements, and the circulation of flyers on professional platforms and groups (LinkedIn). Once the participants were identified for the current study, they were contacted via email by the principal investigator (AJJ). All participants meeting the inclusion criteria provided written informed consent before the interviews.
	Participants were identified and communicated through the investigators (AJJ, ASB, SP)." This is a confusing sentence. Please consider re-writing.	We have rewritten the sentence to improve clarity.	Page: 5<br>Line:19-22<br>Once the participants were identified by the investigators (AJJ, ASB, SP), they were invited by the principal investigator (AJJ) via email to complete the consent form and provide a list of potential dates and

			times, along with their time zones, to schedule the interviews on online platforms (Zoom and Microsoft Teams).
	Reviewer 1: Would it be possible to provide a copy of the interview guide as an appendix?	We appreciate this suggestion and have attached a copy of this to our submission.	Document titled: Interview guide
	Reviewer 1: The initial codes were aimed at addressing the respective research questions of the principal investigator (AJJ).” This is confusing. This is a prospective study, thus, it is unclear what retrospective research question the authors are referring to. Was this study part of another investigation that had different aims?	The suggestion is appreciated, and the lack of clarity is noted. We have removed the sentence.	Page: 6
	Reviewer 2: Data collection was halted after data saturation, as discussed by the research team. No repeat interviews were conducted..’ How did the authors decide when data saturation was achieved?	We appreciate this comment. We have added to the text how the authors concluded that they reached data saturation.	Page: 6<br>Line: 20-23<br>After ten transcripts were coded, the investigators nearly reached data saturation as they started observing repetitive codes and subthemes. The investigators also coded two more transcripts to confirm data saturation and noted that no new themes emerged.
	Reviewer 2: The interview guide was not included in the review. Please add the interview guide for full transparency.	We appreciate this suggestion and have attached a copy of this to our submission.	Document titled: Interview guide
	Reviewer 2: Data analysis: please provide a reference of the used methodology.	The authors agree that we used the methodology of thematic analysis and the appropriate reference has been provided.	Page: 6<br>Line: 11-13<br>A thematic analysis was used in the study. The interviews were audio- and video-recorded, played back, and transcribed offline to ensure the accuracy

			of the information. The verified transcripts were read systematically and analysed using a six-phase thematic analysis(16).
Results	Reviewer 1: Were all the HCPs from adult CR facilities? Were there any pediatric CR experts included? If this was only in adult CR, the authors should clarify that in the methodology.	Thank you for highlighting the patient demographic, much appreciated. We can confirm that our clinicians worked primarily with adults, and this has been clarified in the methodology.	Page: 5<br>Line: 9-11<br>Through purposive sampling, the investigators (AJJ, ASB, SP) identified experts in the field of CR with a minimum of five years of clinical experience with adults who are currently involved in caring for patients recovering from heart disease and major cardiac events.
	Reviewer 1: The theme of kinesiophobia will be elaborated on in detail in this paper.” This is confusing. I am not sure how this sentence contributes to the paper. I was under the impression that this study focused on KP, but after reading this sentence, I went back as I thought I missed something.	This sentence has been removed due to lack of clarity.	
	Reviewer 1: Under “3.6 Subtheme 6: Why is kinesiophobia overlooked?” the second sentence uses ‘FOM’ as an abbreviation for fear of movement, but that abbreviation is not used anywhere else in the paper. In the same respect, there are places throughout the manuscript where cardiac rehabilitation is abbreviated as ‘CR’ and other places where it is written out. Please make consistent.	The comment has been noted and the manuscript has been edited to ensure consistency throughout it.	
	Reviewer 2: Subthemes: please provide more quotes. The authors describe many factors results which are not supported by quotes. It would help of more quotes were provided. The authors refer to the supplementary table which is very helpful	Thank you for this suggestion. We have added additional quotes to the results section for each of the subthemes.	Page: 7<br>Line: 13-25<br><br>Page: 8<br>Line(s): 10-13<br><br>Page: 9<br>Line(s): 1-11<br><br>Page: 10

	but if might help to add some more in the article itself.		Line(s): 1-9<br><br>Page: 10<br>Line(s): 22-25<br><br>Page: 11<br>Line(s): 1-11<br><br>Page: 11<br>Line(s): 21-24<br><br>Page: 12<br>Line(s): 1-11<br><br>Page: 12<br>Line(s): 21-26<br><br>Page: 13<br>Line(s): 1-6
Discussion	Reviewer 1: The participants strongly advised additional training and inclusion of kinesiophobia in the curriculum." What curriculum is this sentence referring to?	The suggestion has been noted and the curricula has been clarified.	Page: 14<br>Line(s): 7-10<br>The participants identified the need to increase awareness of kinesiophobia in professional training, incorporate kinesiophobia in CR curricula for relevant professionals, and increase the representation of psychologists in multidisciplinary teams (MDTs) within CR settings. However, task sharing among professionals does occur (23–26).
Discussion	Reviewer 1: Fundamental awareness of kinesiophobia as fear of movement gained through clinical experience is present among professionals, and the study additionally identified the requirement to include it as part of the educational curriculum of health professionals involved in cardiac rehabilitation. The professionals were not aware of it as an independent phenomenon." What is 'it'?	The need for clarity has been noted and the sentence has been edited.	Page: 13<br>Line(s): 13-15<br>However, awareness of kinesiophobia as an independent construct was variable, as some HCPs perceived it as an expected and valid reaction after a major cardiac event rather than an unusual problem and others viewing it as a separate construct based on severity.

Discussion	Reviewer 1: The current study attempted to understand the HCP's perspective on kinesiophobia under the theme of kinesiophobia." It may be that I am not familiar enough with qualitative research, but this sentence seems confusing. Is it necessary to note that this study on KP uses KP as its theme? It seems like a redundant sentence.	We appreciate the suggestion and have edited the sentence to provide more clarity.	Page: 13<br>Line(s): 8<br>The current study explored HCPs' perspectives on kinesiophobia in the context of CR.
Discussion	Reviewer 1: Sentence: "There seems to be a certain amount of discrepancy regarding the presentation of kinesiophobia in the preliminary stages of CR in the current data and existing literature." Please consider revising this sentence to use more sophisticated language. The authors could revise this sentence to say 'We observed some discrepancy regarding the presentation of….'	The suggestion has been incorporated in the discussion.	Page: 13<br>Line: 23-25<br>The discrepancy noted in the study may be influenced by programme structure, early-phase rehabilitation and the availability of psychological support.
Discussion	Reviewer 1: "Findings that are original to the study are educational and clinical strategies that can be employed to boost the evaluation of kinesiophobia." I believe this study presented HCPs perspectives on KP in CR settings. This sentence is an overreach as I did not find any educational or clinical strategies or recommendations presented by the data collected on how KP can be evaluated.	The suggestions made by the experts have been highlighted in the discussion and substantiated in the results.	Page: 14<br>Line(s): 7-26<br>Page: 15<br>Line(s): 1-2<br>The current study identified specific educational and clinical strategies recommended by participants. The participants identified the need to increase awareness of kinesiophobia in professional training, incorporate kinesiophobia in CR curricula for relevant professionals, and increase the representation of psychologists in multidisciplinary teams (MDTs) within CR settings. However, task sharing among professionals does occur (23–26). The practical implications of this study include increasing awareness

			of kinesiophobia in MDT settings and developing treatment guidelines for non-psychology professionals to empower them to deliver psychologically informed care. CR experts such as nurses, physiotherapists, cardiac electrophysiologists, rehabilitation physicians, exercise physiologists, cardiologists, CR physicians, and cardiac surgeons are involved in formulating and supervising safe and graded levels of physical activity. The CR experts will be well-positioned to apply behavioural strategies to manage kinesiophobia, help patients build confidence to gradually engage in physical activities and exercise, and provide a validating and empathetic environment. (19,20). The clinical management strategies include standardised approaches such as a gentle approach to movement, psychoeducation, family involvement, and the use of objective measures (heart rate monitors, oxygen levels) to build confidence while exercising. The participants also highlighted behavioural strategies such as motivational interviewing, graded exposure, relaxation techniques, and observational learning in group settings.

			HCPs recommended a strong patient-practitioner relationship and collaboration with MDTs. The findings of the present study align with behavioural and psychological strategies supported in other clinical contexts, such as knee arthroplasty and lumbar dysfunction(19,20). The psychological strategies noted in the current study reflect the biopsychosocial model, which emphasises the connection between physical recovery, emotional responses, and social support.
Discussion	Reviewer 1: Lastly, it is clear from the interviews that the HCP were not familiar with KP as an established construct. This should not be surprising as KP originated from the chronic pain research and has not been thoroughly applied in other clinical populations. It would be unreasonable to expect CR HCP to be familiar with psychological constructs from fields outside of their own.	This suggestion is appreciated and we have edited the discussion accordingly.	Page: 7-14
Discussion	Reviewer 2: HCPs also claimed that kinesiophobia was more prevalent during the acute phase of treatment (ICU, in-patient) and lesser in subsequent phases of CR (18). Data suggests that kinesiophobia among patients led to reduced participation in the rehabilitation process and affected prognosis(17).' There is research indicating that kinesiophobia remains stable over time. The authors do not reflect on all the published literature. It might be good to elaborate more on this topic.	We appreciate this comment and have incorporated it in the discussion.	Page: 13<br>Line(s): 21-26<br>The HCPs in the present study reported that kinesiophobia is more prevalent during the acute phase of treatment (ICU, inpatient care) and less common in subsequent phases of CR. Nevertheless, others reported a high level of kinesiophobia over time (18). The discrepancy noted in the study may be influenced by programme structure, early-phase

			rehabilitation and the availability of psychological support. The participants acknowledged that kinesiophobia leads to reduced CR participation and poor prognosis, which aligns with previous evidence(17).
	Reviewer 2:Although standardised guidelines require a psychologist to be part of the multidisciplinary team, it has been found that a psychologist may not always be present, and this potentially impacts how specialists may offer psychological care (22). In identifying practitioners for the study, it was observed that the number of psychologists practicing as part of cardiac rehabilitation was slim.'<br>The authors stress the need for a psychologist. This is indeed important. However, the authors might also want to discuss the role of the physiotherapist in the treatment of kineisophobia.	The suggestion and comment are appreciated. We have incorporated the role of CR experts in the discussion to highlight their roles.	Page: 14<br>Line(s): 13-18<br>CR experts such as nurses, physiotherapists, cardiac electrophysiologists, rehabilitation physicians, exercise physiologists, cardiologists, CR physicians, and cardiac surgeons are involved in formulating and supervising safe and graded levels of physical activity. The CR experts will be well-positioned to apply behavioural strategies to manage kinesiophobia, help patients build confidence to gradually engage in physical activities and exercise, and provide a validating and empathetic environment. (19,20).
Conclusion	Reviewer 1: Yet, physical and psychological strategies were commonly incorporated to facilitate participation in CR." I do not think this is a clear or complete sentence.	The conclusion section has been reedited.	Page: 15<br>Line(s): 18-24

VERSION 2 - REVIEW

Reviewer **2**

Name **Keessen, Paul**

Affiliation **Amsterdam University of Applied Sciences, Faculty of Health**

Date **06-Oct-2025**

COI

I want to congratulate the authors with this interesting study.

Hereby my feedback.

Abstract

1. The exclusion criteria is stated under participants. This seems quite strange.

2. The primary outcome should also include perspectives as this is the title of the study.

3. The conclusion seems very long and some information might be more suitable for the results section.

Strength and limitation

- It also seems that there very few professionals were interviewed.

Discussion

1. Several parts of the discussion might need a bit more reflection. The authors state:

'The participants in the study suggested various physiological and psychological measures used during CR (indicating a biopsychosocial model), but the Tampa Scale for measuring kinesiophobia was mentioned only once'.

Which other tools were suggested? What were the experiences of using these tools?

2. Also, concerning the management of kinesiophobia, the authors do not report studies that investigate exposure based techniques in CR. For example:

- Farris SG, Kibbey MM. Be brave, BE-FIT! A pilot investigation of an ACT-informed exposure intervention to reduce exercise fear-avoidance in older adults. Cogn Behav Ther. 2022 Jul;51(4):273-294. doi: 10.1080/16506073.2022.2037017. Epub 2022 Feb 23. PMID: 35195055; PMCID: PMC8986621.

- Farris SG, Kibbey MM, Keller B, Derby L, Leyro TM, Alderman BL, DiBello AM, Steinberg MB, Abrantes AM. Behavioral Exposure for Interoceptive Tolerance (BE-FIT): A stage II randomized clinical trial protocol. Contemp Clin Trials. 2024 Dec;147:107706. doi: 10.1016/j.cct.2024.107706. Epub 2024 Oct 9. PMID: 39389421.

- https://www.sciencedirect.com/science/article/abs/pii/S1077722924001007

VERSION 2 - AUTHOR RESPONSE

Section	Modification/Clarification requested	Author's comments	Reference
Abstract	Reviewer 2:<br>The exclusion criteria is stated under participants. This seems quite strange.	This has been modified and the sentence removed from the abstract.	Page number: 2
Abstract	Reviewer 2:<br>The primary outcome should also include perspectives as this is the title of the study.	We agree and this change has been incorporated.	Page number: 2<br>Lines: 4-7<br>To explore cardiac rehabilitation (CR) professionals' perspectives on kinesiophobia in patients with cardiovascular diseases. This study aims to understand the perspectives of healthcare professionals (HCPs) regarding their perceptions, assessments, and management of kinesiophobia.
Abstract	Reviewer 2:<br>The conclusion seems very long and some information might be more suitable for the results section.	This was a qualitative study using in-depth interviews which usually does not require large sample sizes like quantitative research. The data collection was terminated once saturation of data was achieved. This has been explained in detail in the methods section of the manuscript.<br><br>The conclusion has been modified according to the feedback.	Page number: 2 & 3<br><br>CR professionals recognise kinesiophobia as a significant issue among patients with heart disease, but do not recognise the term or perceive it as a separate condition; instead, they view it as part of the overall clinical presentation. There is a strong need to advocate for early recognition and assessment of kinesiophobia and for the development of structured management strategies and its inclusion into CR programmes to improve patient outcomes during recovery.
Comments to the author	Reviewer 2:<br>I want to congratulate the authors with this interesting study. Hereby my feedback.	Thank you for your feedback and comments on this study.	
Discussion	Reviewer 2:<br>Several parts of the discussion might need a bit more reflection. The authors state:<br>'The participants in the study suggested various physiological and psychological measures used during CR (indicating a biopsychosocial model), but the Tampa Scale for measuring kinesiophobia was mentioned only once'.<br>Which other tools were suggested? What were the experiences of using these tools?	We did not gather this information as it was not a part of the interview guide and we have therefore modified this sentence. This could be a potential limitation of the study. The following sentence has now been added to the limitations.	Page number: 13 (lines 23 – 25) and 14 (lines 1 – 2).<br>Some of the measures mentioned were the walk test, administration of the Borg scale, measure of heart rate during exercise and exercise capacity, respiratory test, hand dynamometer, Beck's Depression Inventory (BDI), Hospital Anxiety and Depression Scale (HADS), Patient Health Questionnaire-9 (PHQ-9) and Tampa Scale of Kinesiophobia.<br><br>Page number: 15<br>Line: 23 – 24

			Information on tools for assessment of kinesiophobia and specific interventions were not explored in detail.
Discussion	Reviewer 2:<br>Also, concerning the management of kinesiophobia, the authors do not report studies that investigate exposure based techniques in CR.	Thank you for the additional references. These have been incorporated into the discussion.	Page number: 15<br>Line: 5 – 7<br>Recent Behavioural Exposure for Interoceptive Tolerance (BE-FIT), an ACT-informed exposure-based intervention focuses on accepting bodily sensations rather than eliminating fear, appears to hold promise as a potential strategy.
